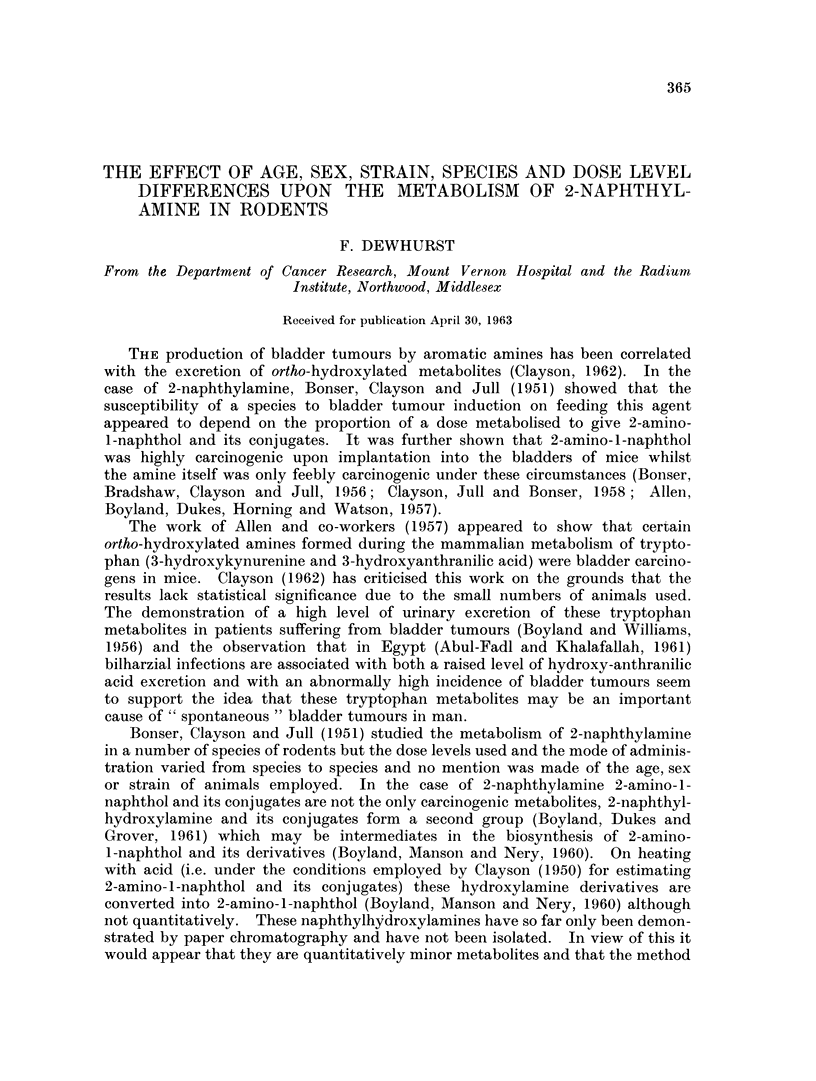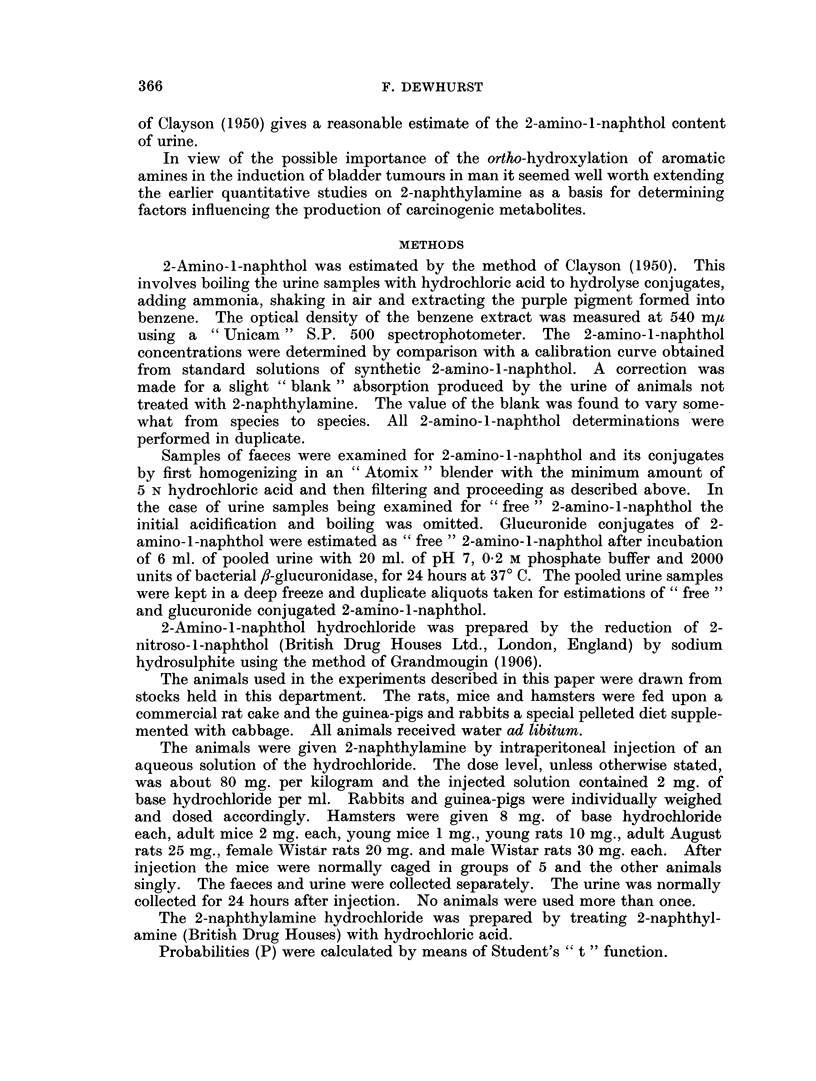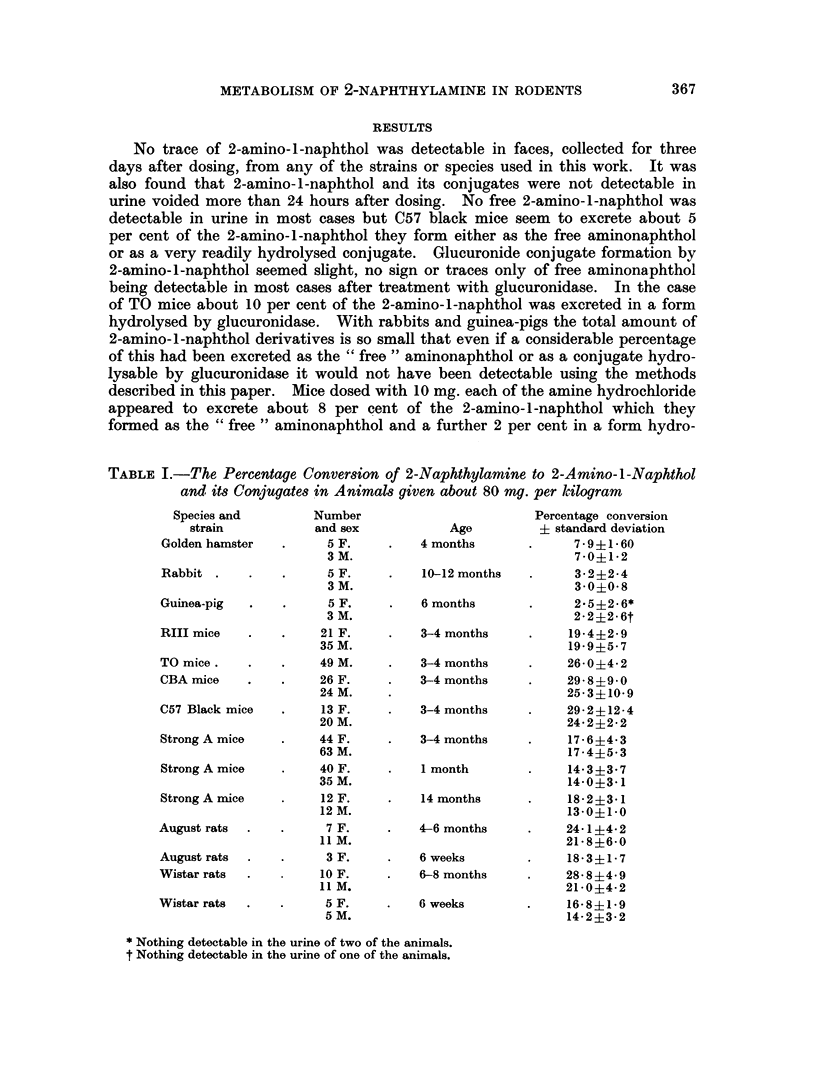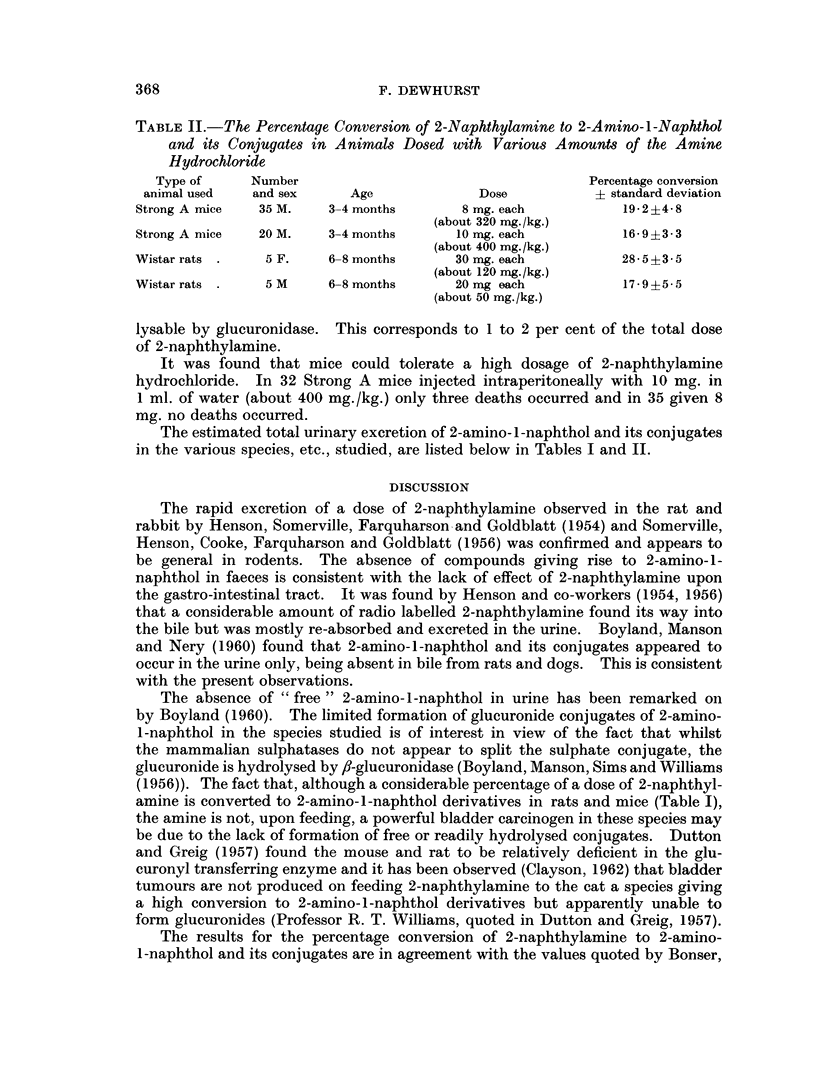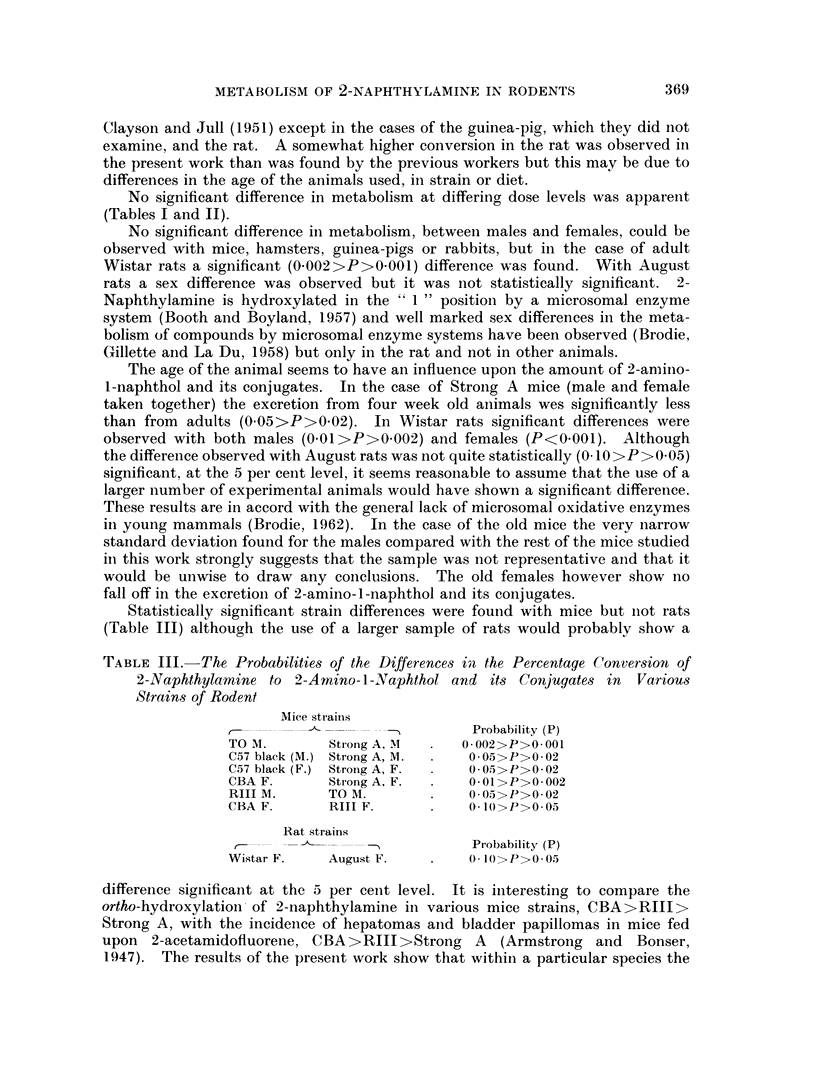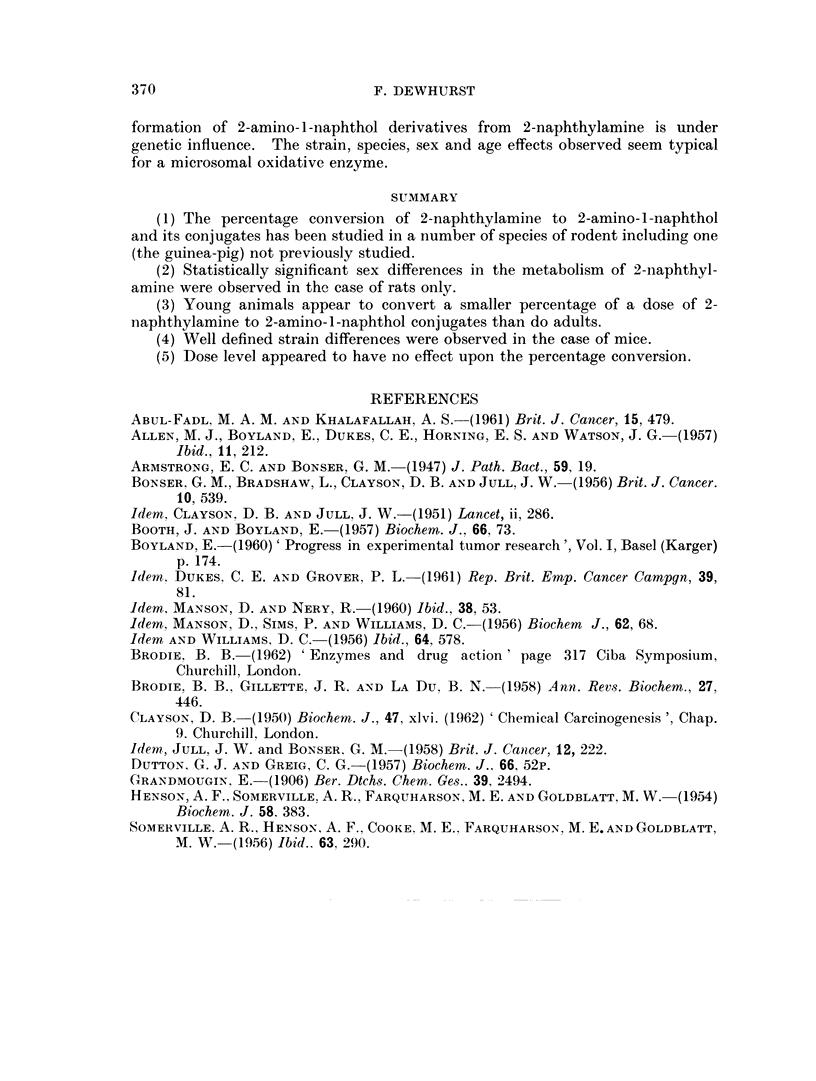# The Effect of Age, Sex, Strain, Species and Dose Level Differences Upon the Metabolism of 2-Naphthylamine in Rodents

**DOI:** 10.1038/bjc.1963.50

**Published:** 1963-06

**Authors:** F. Dewhurst


### THE EFFECT OF AGE, SEX, STRAIN, SPECIES AND DOSE LEVEL DIFFERENCES UPON THE METABOLISM OF 2-NAPHTHYLAMINE IN RODENTS

F. DEWHURST

*From the Department of Cancer Research, Mount Vernon Hospital and the Radium Institute, Northwood, Middlesex*

Received for publication April 30, 1963

THE production of bladder tumours by aromatic amines has been correlated with the excretion of *ortho*-hydroxylated metabolites (Clayson, 1962). In the case of 2-naphthylamine, Bonser, Clayson and Jull (1951) showed that the susceptibility of a species to bladder tumour induction on feeding this agent appeared to depend on the proportion of a dose metabolised to give 2-amino-1-naphthol and its conjugates. It was further shown that 2-amino-1-naphthol was highly carcinogenic upon implantation into the bladders of mice whilst the amine itself was only feebly carcinogenic under these circumstances (Bonser, Bradshaw, Clayson and Jull, 1956; Clayson, Jull and Bonser, 1958; Allen, Boyland, Dukes, Horning and Watson, 1957).

The work of Allen and co-workers (1957) appeared to show that certain *ortho*-hydroxylated amines formed during the mammalian metabolism of tryptophan (3-hydroxykynurenine and 3-hydroxyanthranilic acid) were bladder carcinogens in mice. Clayson (1962) has criticised this work on the grounds that the results lack statistical significance due to the small numbers of animals used. The demonstration of a high level of urinary excretion of these tryptophan metabolites in patients suffering from bladder tumours (Boyland and Williams, 1956) and the observation that in Egypt (Abul-Fadl and Khalafallah, 1961) bilharzial infections are associated with both a raised level of hydroxy-anthranilic acid excretion and with an abnormally high incidence of bladder tumours seem to support the idea that these tryptophan metabolites may be an important cause of " spontaneous " bladder tumours in man.

Bonser, Clayson and Jull (1951) studied the metabolism of 2-naphthylamine in a number of species of rodents but the dose levels used and the mode of administration varied from species to species and no mention was made of the age, sex or strain of animals employed. In the case of 2-naphthylamine 2-amino-1-naphthol and its conjugates are not the only carcinogenic metabolites, 2-naphthylhydroxylamine and its conjugates form a second group (Boyland, Dukes and Grover, 1961) which may be intermediates in the biosynthesis of 2-amino-1-naphthol and its derivatives (Boyland, Manson and Nery, 1960). On heating with acid (i.e. under the conditions employed by Clayson (1950) for estimating 2-amino-1-naphthol and its conjugates) these hydroxylamine derivatives are converted into 2-amino-1-naphthol (Boyland, Manson and Nery, 1960) although not quantitatively. These naphthylhydroxylamines have so far only been demonstrated by paper chromatography and have not been isolated. In view of this it would appear that they are quantitatively minor metabolites and that the method of Clayson (1950) gives a reasonable estimate of the 2-amino-1-naphthol content of urine.

In view of the possible importance of the *ortho*-hydroxylation of aromatic amines in the induction of bladder tumours in man it seemed well worth extending the earlier quantitative studies on 2-naphthylamine as a basis for determining factors influencing the production of carcinogenic metabolites.

### METHODS

2-Amino-1-naphthol was estimated by the method of Clayson (1950). This involves boiling the urine samples with hydrochloric acid to hydrolyse conjugates, adding ammonia, shaking in air and extracting the purple pigment formed into benzene. The optical density of the benzene extract was measured at 540 m$\mu$ using a "Unicam" S.P. 500 spectrophotometer. The 2-amino-1-naphthol concentrations were determined by comparison with a calibration curve obtained from standard solutions of synthetic 2-amino-1-naphthol. A correction was made for a slight "blank" absorption produced by the urine of animals not treated with 2-naphthylamine. The value of the blank was found to vary somewhat from species to species. All 2-amino-1-naphthol determinations were performed in duplicate.

Samples of faeces were examined for 2-amino-1-naphthol and its conjugates by first homogenizing in an "Atomix" blender with the minimum amount of 5 N hydrochloric acid and then filtering and proceeding as described above. In the case of urine samples being examined for "free" 2-amino-1-naphthol the initial acidification and boiling was omitted. Glucuronide conjugates of 2-amino-1-naphthol were estimated as "free" 2-amino-1-naphthol after incubation of 6 ml. of pooled urine with 20 ml. of pH 7, 0·2 M phosphate buffer and 2000 units of bacterial $\beta$-glucuronidase, for 24 hours at 37° C. The pooled urine samples were kept in a deep freeze and duplicate aliquots taken for estimations of "free" and glucuronide conjugated 2-amino-1-naphthol.

2-Amino-1-naphthol hydrochloride was prepared by the reduction of 2-nitroso-1-naphthol (British Drug Houses Ltd., London, England) by sodium hydrosulphite using the method of Grandmougin (1906).

The animals used in the experiments described in this paper were drawn from stocks held in this department. The rats, mice and hamsters were fed upon a commercial rat cake and the guinea-pigs and rabbits a special pelleted diet supplemented with cabbage. All animals received water *ad libitum.*

The animals were given 2-naphthylamine by intraperitoneal injection of an aqueous solution of the hydrochloride. The dose level, unless otherwise stated, was about 80 mg. per kilogram and the injected solution contained 2 mg. of base hydrochloride per ml. Rabbits and guinea-pigs were individually weighed and dosed accordingly. Hamsters were given 8 mg. of base hydrochloride each, adult mice 2 mg. each, young mice 1 mg., young rats 10 mg., adult August rats 25 mg., female Wistar rats 20 mg. and male Wistar rats 30 mg. each. After injection the mice were normally caged in groups of 5 and the other animals singly. The faeces and urine were collected separately. The urine was normally collected for 24 hours after injection. No animals were used more than once.

The 2-naphthylamine hydrochloride was prepared by treating 2-naphthylamine (British Drug Houses) with hydrochloric acid.

Probabilities (P) were calculated by means of Student's "t" function.

### RESULTS

No trace of 2-amino-1-naphthol was detectable in faces, collected for three days after dosing, from any of the strains or species used in this work. It was also found that 2-amino-1-naphthol and its conjugates were not detectable in urine voided more than 24 hours after dosing. No free 2-amino-1-naphthol was detectable in urine in most cases but C57 black mice seem to excrete about 5 per cent of the 2-amino-1-naphthol they form either as the free aminonaphthol or as a very readily hydrolysed conjugate. Glucuronide conjugate formation by 2-amino-1-naphthol seemed slight, no sign or traces only of free aminonaphthol being detectable in most cases after treatment with glucuronidase. In the case of TO mice about 10 per cent of the 2-amino-1-naphthol was excreted in a form hydrolysed by glucuronidase. With rabbits and guinea-pigs the total amount of 2-amino-1-naphthol derivatives is so small that even if a considerable percentage of this had been excreted as the " free " aminonaphthol or as a conjugate hydrolysable by glucuronidase it would not have been detectable using the methods described in this paper. Mice dosed with 10 mg. each of the amine hydrochloride appeared to excrete about 8 per cent of the 2-amino-1-naphthol which they formed as the " free " aminonaphthol and a further 2 per cent in a form hydro-

TABLE I.—*The Percentage Conversion of 2-Naphthylamine to 2-Amino-1-Naphthol and its Conjugates in Animals given about 80 mg. per kilogram*

| Species and strain | Number and sex | Age | Percentage conversion ± standard deviation |
|---|---|---|---|
| Golden hamster | 5 F. | 4 months | 7·9±1·60 |
| | 3 M. | | 7·0±1·2 |
| Rabbit | 5 F. | 10–12 months | 3·2±2·4 |
| | 3 M. | | 3·0±0·8 |
| Guinea-pig | 5 F. | 6 months | 2·5±2·6* |
| | 3 M. | | 2·2±2·6† |
| RIII mice | 21 F. | 3–4 months | 19·4±2·9 |
| | 35 M. | | 19·9±5·7 |
| TO mice | 49 M. | 3–4 months | 26·0±4·2 |
| CBA mice | 26 F. | 3–4 months | 29·8±9·0 |
| | 24 M. | | 25·3±10·9 |
| C57 Black mice | 13 F. | 3–4 months | 29·2±12·4 |
| | 20 M. | | 24·2±2·2 |
| Strong A mice | 44 F. | 3–4 months | 17·6±4·3 |
| | 63 M. | | 17·4±5·3 |
| Strong A mice | 40 F. | 1 month | 14·3±3·7 |
| | 35 M. | | 14·0±3·1 |
| Strong A mice | 12 F. | 14 months | 18·2±3·1 |
| | 12 M. | | 13·0±1·0 |
| August rats | 7 F. | 4–6 months | 24·1±4·2 |
| | 11 M. | | 21·8±6·0 |
| August rats | 3 F. | 6 weeks | 18·3±1·7 |
| Wistar rats | 10 F. | 6–8 months | 28·8±4·9 |
| | 11 M. | | 21·0±4·2 |
| Wistar rats | 5 F. | 6 weeks | 16·8±1·9 |
| | 5 M. | | 14·2±3·2 |

* Nothing detectable in the urine of two of the animals.
† Nothing detectable in the urine of one of the animals.

TABLE II.—*The Percentage Conversion of 2-Naphthylamine to 2-Amino-1-Naphthol and its Conjugates in Animals Dosed with Various Amounts of the Amine Hydrochloride*

| Type of animal used | Number and sex | Age | Dose | Percentage conversion ± standard deviation |
|---|---|---|---|---|
| Strong A mice | 35 M. | 3–4 months | 8 mg. each (about 320 mg./kg.) | 19·2±4·8 |
| Strong A mice | 20 M. | 3–4 months | 10 mg. each (about 400 mg./kg.) | 16·9±3·3 |
| Wistar rats . | 5 F. | 6–8 months | 30 mg. each (about 120 mg./kg.) | 28·5±3·5 |
| Wistar rats . | 5 M | 6–8 months | 20 mg each (about 50 mg./kg.) | 17·9±5·5 |

lysable by glucuronidase. This corresponds to 1 to 2 per cent of the total dose of 2-naphthylamine.

It was found that mice could tolerate a high dosage of 2-naphthylamine hydrochloride. In 32 Strong A mice injected intraperitoneally with 10 mg. in 1 ml. of water (about 400 mg./kg.) only three deaths occurred and in 35 given 8 mg. no deaths occurred.

The estimated total urinary excretion of 2-amino-1-naphthol and its conjugates in the various species, etc., studied, are listed below in Tables I and II.

### DISCUSSION

The rapid excretion of a dose of 2-naphthylamine observed in the rat and rabbit by Henson, Somerville, Farquharson and Goldblatt (1954) and Somerville, Henson, Cooke, Farquharson and Goldblatt (1956) was confirmed and appears to be general in rodents. The absence of compounds giving rise to 2-amino-1-naphthol in faeces is consistent with the lack of effect of 2-naphthylamine upon the gastro-intestinal tract. It was found by Henson and co-workers (1954, 1956) that a considerable amount of radio labelled 2-naphthylamine found its way into the bile but was mostly re-absorbed and excreted in the urine. Boyland, Manson and Nery (1960) found that 2-amino-1-naphthol and its conjugates appeared to occur in the urine only, being absent in bile from rats and dogs. This is consistent with the present observations.

The absence of " free " 2-amino-1-naphthol in urine has been remarked on by Boyland (1960). The limited formation of glucuronide conjugates of 2-amino-1-naphthol in the species studied is of interest in view of the fact that whilst the mammalian sulphatases do not appear to split the sulphate conjugate, the glucuronide is hydrolysed by $\beta$-glucuronidase (Boyland, Manson, Sims and Williams (1956)). The fact that, although a considerable percentage of a dose of 2-naphthylamine is converted to 2-amino-1-naphthol derivatives in rats and mice (Table I), the amine is not, upon feeding, a powerful bladder carcinogen in these species may be due to the lack of formation of free or readily hydrolysed conjugates. Dutton and Greig (1957) found the mouse and rat to be relatively deficient in the glucuronyl transferring enzyme and it has been observed (Clayson, 1962) that bladder tumours are not produced on feeding 2-naphthylamine to the cat a species giving a high conversion to 2-amino-1-naphthol derivatives but apparently unable to form glucuronides (Professor R. T. Williams, quoted in Dutton and Greig, 1957).

The results for the percentage conversion of 2-naphthylamine to 2-amino-1-naphthol and its conjugates are in agreement with the values quoted by Bonser,

Clayson and Jull (1951) except in the cases of the guinea-pig, which they did not examine, and the rat. A somewhat higher conversion in the rat was observed in the present work than was found by the previous workers but this may be due to differences in the age of the animals used, in strain or diet.

No significant difference in metabolism at differing dose levels was apparent (Tables I and II).

No significant difference in metabolism, between males and females, could be observed with mice, hamsters, guinea-pigs or rabbits, but in the case of adult Wistar rats a significant ($0{\cdot}002 > P > 0{\cdot}001$) difference was found. With August rats a sex difference was observed but it was not statistically significant. 2-Naphthylamine is hydroxylated in the " 1 " position by a microsomal enzyme system (Booth and Boyland, 1957) and well marked sex differences in the metabolism of compounds by microsomal enzyme systems have been observed (Brodie, Gillette and La Du, 1958) but only in the rat and not in other animals.

The age of the animal seems to have an influence upon the amount of 2-amino-1-naphthol and its conjugates. In the case of Strong A mice (male and female taken together) the excretion from four week old animals wes significantly less than from adults ($0{\cdot}05 > P > 0{\cdot}02$). In Wistar rats significant differences were observed with both males ($0{\cdot}01 > P > 0{\cdot}002$) and females ($P < 0{\cdot}001$). Although the difference observed with August rats was not quite statistically ($0{\cdot}10 > P > 0{\cdot}05$) significant, at the 5 per cent level, it seems reasonable to assume that the use of a larger number of experimental animals would have shown a significant difference. These results are in accord with the general lack of microsomal oxidative enzymes in young mammals (Brodie, 1962). In the case of the old mice the very narrow standard deviation found for the males compared with the rest of the mice studied in this work strongly suggests that the sample was not representative and that it would be unwise to draw any conclusions. The old females however show no fall off in the excretion of 2-amino-1-naphthol and its conjugates.

Statistically significant strain differences were found with mice but not rats (Table III) although the use of a larger sample of rats would probably show a

TABLE III.—*The Probabilities of the Differences in the Percentage Conversion of 2-Naphthylamine to 2-Amino-1-Naphthol and its Conjugates in Various Strains of Rodent*

| Mice strains | | Probability (P) |
|---|---|---|
| TO M. | Strong A, M | $0{\cdot}002 > P > 0{\cdot}001$ |
| C57 black (M.) | Strong A, M. | $0{\cdot}05 > P > 0{\cdot}02$ |
| C57 black (F.) | Strong A, F. | $0{\cdot}05 > P > 0{\cdot}02$ |
| CBA F. | Strong A, F. | $0{\cdot}01 > P > 0{\cdot}002$ |
| RIII M. | TO M. | $0{\cdot}05 > P > 0{\cdot}02$ |
| CBA F. | RIII F. | $0{\cdot}10 > P > 0{\cdot}05$ |

| Rat strains | | Probability (P) |
|---|---|---|
| Wistar F. | August F. | $0{\cdot}10 > P > 0{\cdot}05$ |

difference significant at the 5 per cent level. It is interesting to compare the *ortho*-hydroxylation of 2-naphthylamine in various mice strains, CBA > RIII > Strong A, with the incidence of hepatomas and bladder papillomas in mice fed upon 2-acetamidofluorene, CBA > RIII > Strong A (Armstrong and Bonser, 1947). The results of the present work show that within a particular species the formation of 2-amino-1-naphthol derivatives from 2-naphthylamine is under genetic influence. The strain, species, sex and age effects observed seem typical for a microsomal oxidative enzyme.

### SUMMARY

(1) The percentage conversion of 2-naphthylamine to 2-amino-1-naphthol and its conjugates has been studied in a number of species of rodent including one (the guinea-pig) not previously studied.

(2) Statistically significant sex differences in the metabolism of 2-naphthylamine were observed in the case of rats only.

(3) Young animals appear to convert a smaller percentage of a dose of 2-naphthylamine to 2-amino-1-naphthol conjugates than do adults.

(4) Well defined strain differences were observed in the case of mice.

(5) Dose level appeared to have no effect upon the percentage conversion.

### REFERENCES

ABUL-FADL, M. A. M. AND KHALAFALLAH, A. S.—(1961) *Brit. J. Cancer*, **15**, 479.

ALLEN, M. J., BOYLAND, E., DUKES, C. E., HORNING, E. S. AND WATSON, J. G.—(1957) *Ibid.*, **11**, 212.

ARMSTRONG, E. C. AND BONSER, G. M.—(1947) *J. Path. Bact.*, **59**, 19.

BONSER, G. M., BRADSHAW, L., CLAYSON, D. B. AND JULL, J. W.—(1956) *Brit. J. Cancer*. **10**, 539.

*Idem*, CLAYSON, D. B. AND JULL, J. W.—(1951) *Lancet*, ii, 286.

BOOTH, J. AND BOYLAND, E.—(1957) *Biochem. J.*, **66**, 73.

BOYLAND, E.—(1960) ' Progress in experimental tumor research ', Vol. I, Basel (Karger) p. 174.

*Idem*, DUKES, C. E. AND GROVER, P. L.—(1961) *Rep. Brit. Emp. Cancer Campgn*, **39**, 81.

*Idem*, MANSON, D. AND NERY, R.—(1960) *Ibid.*, **38**, 53.

*Idem*, MANSON, D., SIMS, P. AND WILLIAMS, D. C.—(1956) *Biochem J.*, **62**, 68.

*Idem* AND WILLIAMS, D. C.—(1956) *Ibid.*, **64**, 578.

BRODIE, B. B.—(1962) ' Enzymes and drug action ' page 317 Ciba Symposium, Churchill, London.

BRODIE, B. B., GILLETTE, J. R. AND LA DU, B. N.—(1958) *Ann. Revs. Biochem.*, **27**, 446.

CLAYSON, D. B.—(1950) *Biochem. J.*, **47**, xlvi. (1962) ' Chemical Carcinogenesis ', Chap. 9, Churchill, London.

*Idem*, JULL, J. W. and BONSER, G. M.—(1958) *Brit. J. Cancer*, **12**, 222.

DUTTON, G. J. AND GREIG, C. G.—(1957) *Biochem. J.*, **66**, 52P.

GRANDMOUGIN, E.—(1906) *Ber. Dtchs. Chem. Ges.*, **39**, 2494.

HENSON, A. F., SOMERVILLE, A. R., FARQUHARSON, M. E. AND GOLDBLATT, M. W.—(1954) *Biochem. J.* **58**, 383.

SOMERVILLE, A. R., HENSON, A. F., COOKE, M. E., FARQUHARSON, M. E. AND GOLDBLATT, M. W.—(1956) *Ibid.*, **63**, 290.